# Population differences and domestication effects on mating and remating frequencies in Queensland fruit fly

**DOI:** 10.1038/s41598-021-04198-4

**Published:** 2022-01-07

**Authors:** Khandaker Asif Ahmed, Heng Lin Yeap, Gunjan Pandey, Siu Fai Lee, Phillip W. Taylor, John G. Oakeshott

**Affiliations:** 1 Applied BioSciences, Macquarie University, Macquarie Park, NSW 2109 Australia; 2 CSIRO Land and Water, Black Mountain, ACT 2601 Australia

**Keywords:** Sexual selection, Animal behaviour, Evolutionary biology, Genetic variation

## Abstract

Females of many insect species are unreceptive to remating for a period following their first mating. This inhibitory effect may be mediated by either the female or her first mate, or both, and often reflects the complex interplay of reproductive strategies between the sexes. Natural variation in remating inhibition and how this phenotype responds to captive breeding are largely unexplored in insects, including many pest species. We investigated genetic variation in remating propensity in the Queensland fruit fly, *Bactrocera tryoni*, using strains differing in source locality and degree of domestication. We found up to threefold inherited variation between strains from different localities in the level of intra-strain remating inhibition. The level of inhibition also declined significantly during domestication, which implied the existence of genetic variation for this trait within the starting populations as well. Inter-strain mating and remating trials showed that the strain differences were mainly due to the genotypes of the female and, to a lesser extent, the second male, with little effect of the initial male genotype. Implications for our understanding of fruit fly reproductive biology and population genetics and the design of Sterile Insect Technique pest management programs are discussed.

## Introduction

Polyandry in many insect species entails complex and often conflicting reproductive strategies both within and between the sexes. One common outcome is that females become refractory to remating for a period after an initial mating^1,2^. This may be promoted by females to avoid fitness costs from unnecessary copulation or by the first mating males to promote the use of their sperm in fertilising eggs^1,3–5^. Depending on demographic factors, however, such remating inhibition also has potential disadvantages; for example, a once-mated female may later have insufficient stored sperm to fertilise all her eggs, may only have a poor-quality male’s sperm available, and may be disadvantaged by low genetic diversity amongst her offspring. Moreover the optimal strategy for each sex will often impact that of the other sex; for example, while effective remating inhibition by a female’s first mate may maximise her use of his sperm, it may also mean that she runs out of sperm and then be unable to reproduce without further mating^1^. This has led to much debate about the consequences for genetic variances and covariances contributing to mating behaviours^6–8^. However, empirical data on the issue has often been difficult to obtain.

The best characterised systems to date are from drosophilids, in particular *Drosophila melanogaster*^3,4,9^, in which mated females exhibit weak remating inhibition for the first few hours, followed by a stronger form of inhibition which may last for more than a week^10–12^. Social factors such as population density and frequency of exposure to males influence female remating propensity in *D. melanogaster* and females may be more likely to remate with males from their own strain than from other strains^13–16^. However, genetic differences in remating inhibition have also been described, both between wild populations and between wild and domesticated strains^8,17–20^. Inherited responses to selection have also been described in strains on which different mating regime (e.g. opportunities for each sex to mate) have been imposed^7^. The genotype of the female can be important, as can be the genotype of both the first and subsequent males^21,22^. However specific genes responsible for the differences have seldom been identified.

Males’ influence on female remating in *D. melanogaster* is largely mediated by the quality and quantity of the sperm and certain of the > 200 seminal fluid proteins (SFPs) identified so far^23–28^. These proteins are mainly produced in the male accessory glands, with some also produced in ejaculatory tissue. Three specific SFPs, the Sex Peptide (SP)^29–31^, Dup99B^32^, and Esterase 6^33,34^, have been shown to contribute directly to remating inhibition and, of these, the SP is best characterised at a mechanistic level^35–38^. Initially, short-term inhibition (the first few hours post-mating) is induced by free SP, with longer term inhibition (~ a week) resulting from the gradual release of SP bound to sperm tails^39^. Intriguingly, SP and its homologues have so far only been found in one small lineage of the genus *Drosophila*, although homologues of the SP receptor are much more widely distributed^37^. The taxonomic distribution of SP-based mechanisms is thus unclear.

Female remating inhibition has also been observed, and characterised to some degree, in several pest species of tephritid fruit fly^40–47^. This is in part because of its crucial role in the Sterile Insect Technique (SIT) which is increasingly used to control some of these species. Population suppression via SIT depends on field females that have mated with mass released sterile males having low receptivity to remating with fertile field males^48,49^, so much of the research has focussed on male-derived effects on the inhibition^47,50^. Somewhat akin to the situation in *D. melanogaster*, sperm storage induces an initial, short term refractiveness and accessory gland products induce longer term inhibition in the Mediterranean fruit fly, *Ceratitis capitata*^51^. However, an effect of sperm storage has not yet been found in two other tephritid genera, *Bactrocera* and *Anastrepha*^52–56^. We are unaware of any previous surveys for population differences in remating inhibition in tephritids but differences in remating inhibition between females from undomesticated and domesticated strains have been described in the Mexican fruit fly, *A. ludens*. Specifically, lower levels of remating inhibition have been found in domesticated females than wild females when they are first mated with either undomesticated or domesticated males^57^*.*

As with other studied *Bactrocera* species, the Queensland fruit fly (Qfly), *B. tryoni*, exhibits a seminal fluid induced male effect on female remating inhibition, with no evidence as yet of sperm effects and equivocal evidence thus far for female effects^47,54,58–62^. Similar to *A. ludens*, changes in the level of remating inhibition might be expected during Qfly domestication, because domestication effects have been described for other reproductive behaviours and the (nutrient-dependent) development of both sexes’ reproductive organs^61,62^. However, a recent comparison of old (Generation 49) and young (Generation 5) Qfly colonies from Brisbane, Australia, found similar levels of remating inhibition in all combinations of males and females from the two colonies^62^.

The present study first compares the mating and remating propensities of recently established Qfly colonies from three different field populations and traces changes in those traits during domestication. Having found heritable differences in remating propensities between populations and during domestication, intra- and inter-strain pairing experiments are then used to evaluate the respective contributions of males and females to the observed differences.

## Results

### Experiment 1: effects of source population and domestication

This experiment screened for genetic differences in mating and remating frequencies between Qfly populations and tested whether those frequencies changed during domestication. Three field populations were sampled, one from a tropical region (Cape Tribulation; CT) and two from temperate regions (Sydney and Canberra; SYD and CBR respectively). Two collections were made several months apart in each of the latter populations (SYD-1 and SYD-2 and CBR-1 and CBR-2 for the first and second collections respectively). Each population was tested at five or six generations (two-four generations for each of the two strains from each of SYD and CBR) across their first 15–20 generations in the laboratory. A long domesticated control strain (S06)^63^, originally collected from Sydney, was also tested six times between generations ~ 116–126.

**Figure 1:**
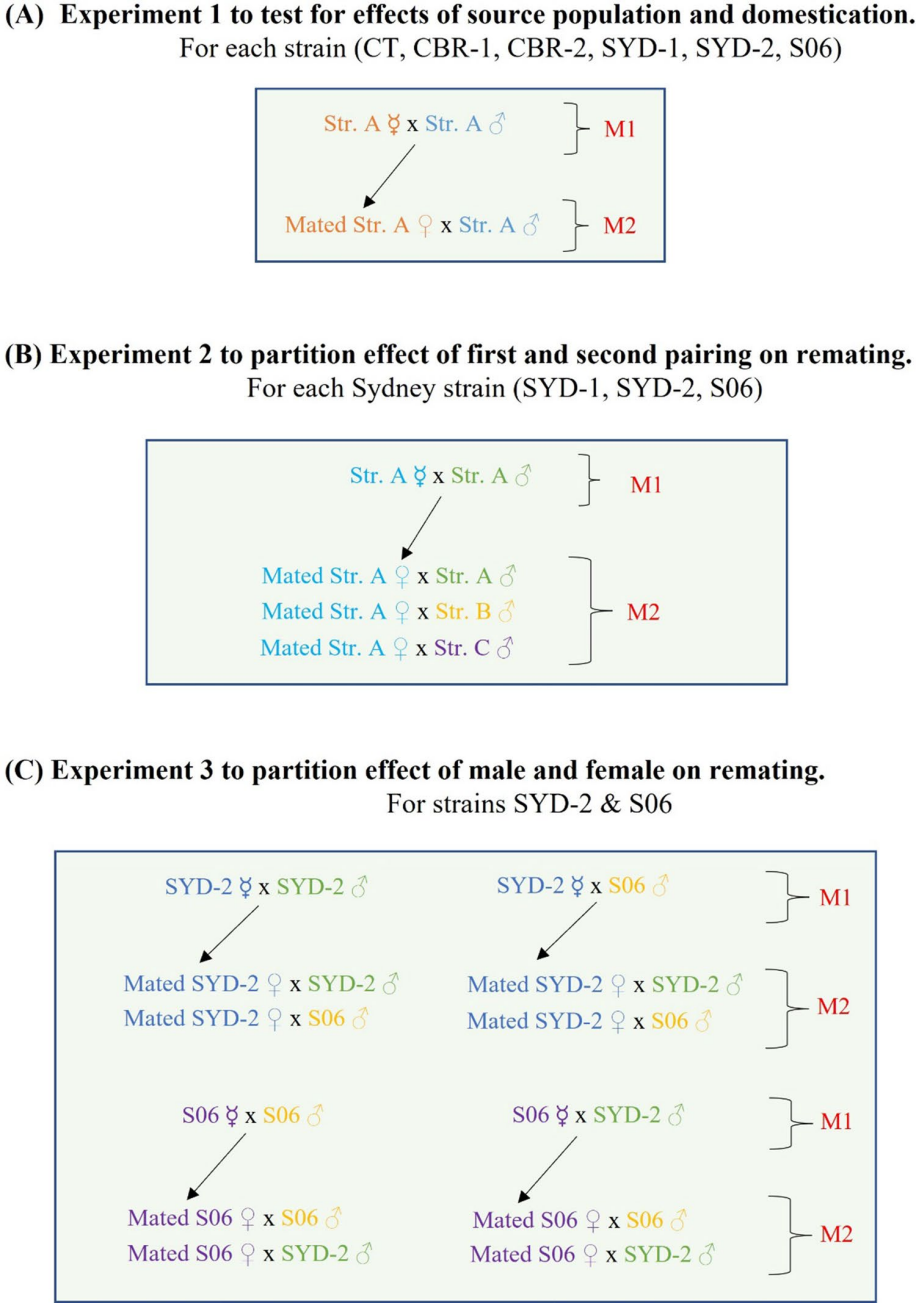
Schematic representation of the mating and remating schemes for the three Experiments. Panels **(A)**, **(B)** and **(C)** cover Experiments 1, 2 and 3 respectively.

Mating and remating frequencies were assessed from individual intra-strain pairings of age-synchronised flies (Fig. 1A and see Materials and methods). Two cohorts of virgin females were used for each strain-generation combination tested. The first cohort of virgin females were paired with virgin males over a period of about two and half hours before the onset of simulated dusk (Qfly mating is closely confined to dusk^64–66^) and those that had mated in the initial pairing were then held for a further four days without exposure to males. Both cohorts of females were then paired with a second set of virgin males using the same protocol. We estimated first mating frequency (M1%) from the second cohort and remating frequency (M2%) from the first cohort in the analyses below because they were obtained on the same day of testing. (Occasionally differences were found from the M1% data for the first cohort, i.e., collected on flies 4 days younger and on a different testing day; Table S1).

**Figure 2:**
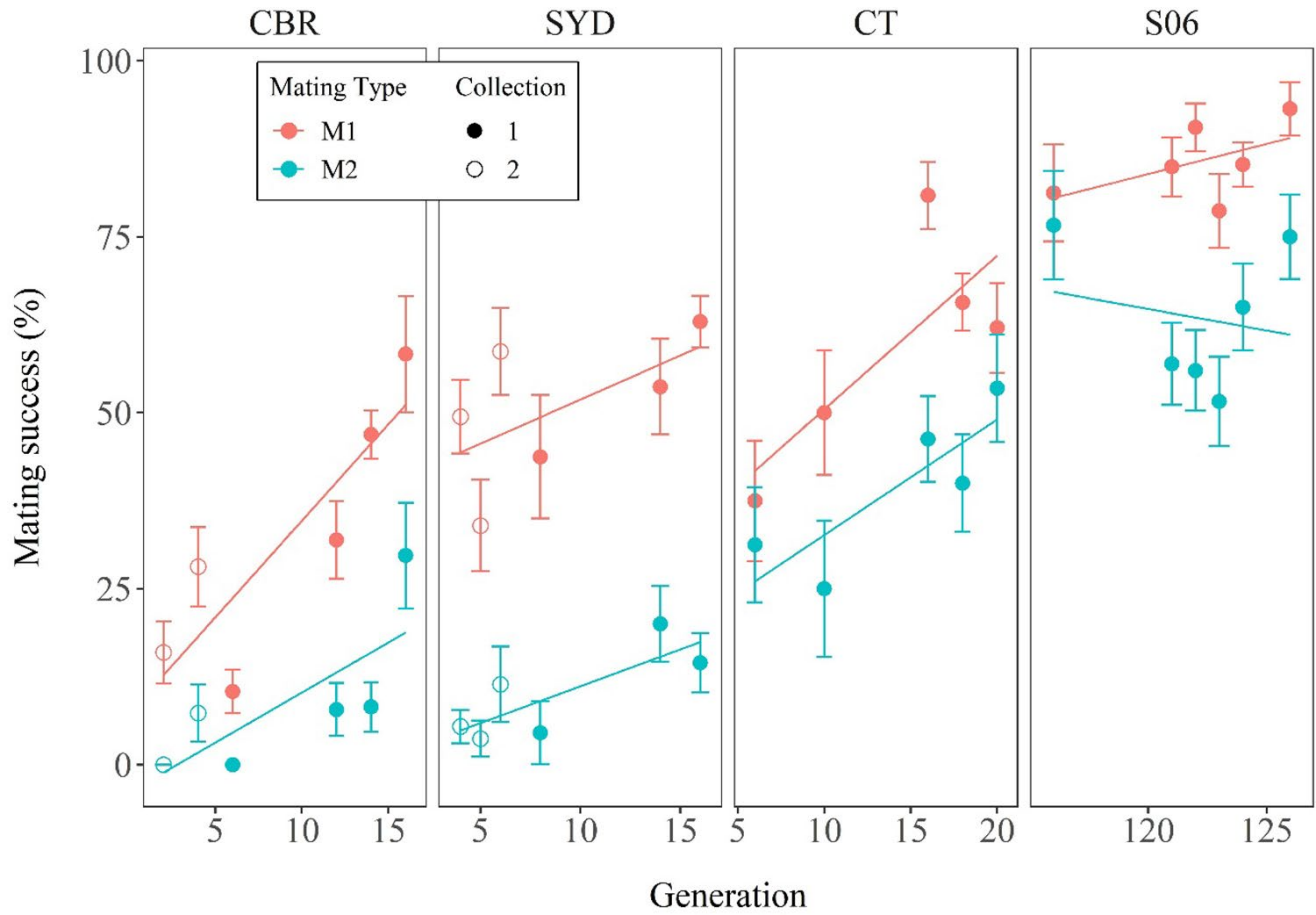
Changes in the mating and remating frequencies of the three wild/caught populations and S06 over generations in Experiment 1. The data for the -1 and -2 collections of CBR and SYD are shown with filled and open symbols respectively. Standard errors are also shown. Lines of best fit for each population across generations are given for both M1% (red) and M2% (blue). Numbers of females tested per strain and generation were 32–211 for M1 and 30–92 for M2.

M1% varied significantly between the three populations and increased over the course of domestication in each population, rising from < 20% in CBR in the first few generations tested (G2-G4) to > 70% in CT in the last few generations tested (G16-G20) (Fig. 2). Values for S06 were higher still, ~ 85–93%, with no significant trend over time. Analysis of deviance of a quasibinomial generalized linear model (GLM, with dispersion parameter 2.89) showed highly significant effects of both population (χ^2^ = 14.96, df = 2, *p* <  < 0.001) and the natural logarithm of generation number (χ^2^ = 15.66, df = 1, *p* <  < 0.001). The data for the two strains from SYD and the two strains from CBR showed the same patterns of population and generation differences (Fig. 2), suggesting the two strains were representative of their respective source population in each case. The major difference between the populations was the relatively low M1% for CBR.

M2%s were always lower than the corresponding M1%s, confirming that some level of remating inhibition was universal across the strains and generations tested (Fig. 2). In absolute terms (i.e., the difference between M2% and M1%) the difference was as little as ~ 10–15% in CT and S06 but as much as ~ 40% in CBR and SYD, indicating systematic strain variation in the level of inhibition.

However, many other aspects of the M2% results were similar to those for M1%. Once again there was a general trend for values to increase over generations, albeit remaining lower than those for S06 even at the last generation scored, with S06 showing no consistent change over time. Analysis of deviance of a quasibinomial model (dispersion parameter 1.56) again confirmed statistically significant effects of population (χ^2^ = 29.41, df = 2, *p* <  < 0.001) and natural logarithm of generation number (χ^2^ = 18.89, df = 1, *p* <  < 0.001). And again the data were consistent between the two SYD strains and between the two CBR strains.

**Figure 3:**
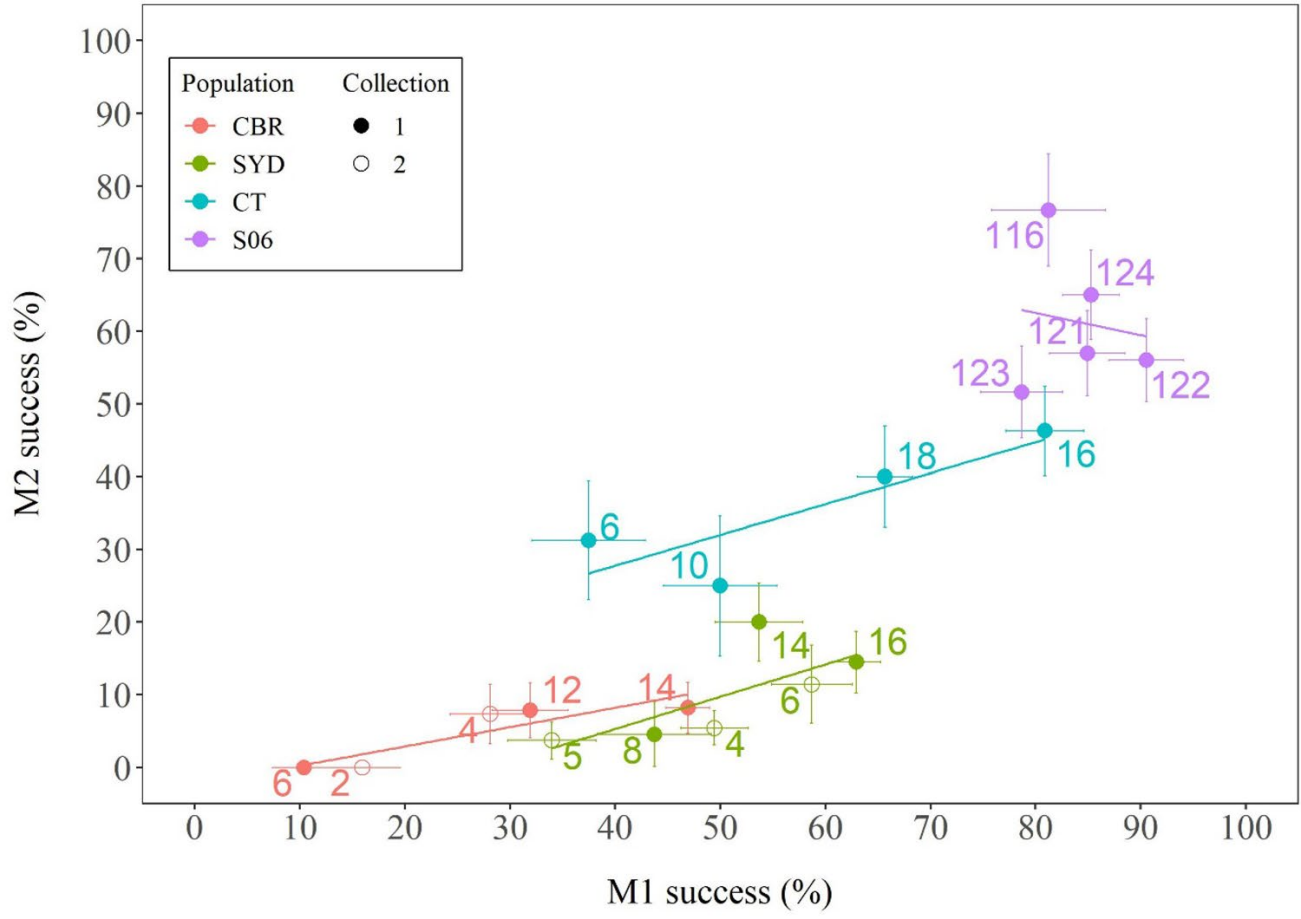
The correlation between M1% and M2% across all source population-generation combinations in Experiment 1. Data are taken from Fig. 2 with populations shown in different colours. The data for the -1 and -2 collections of CBR and SYD are shown with filled and open symbols respectively and generations are indicated by the numbers adjacent each symbol. Lines of best fit are shown for each population.

**Figure 4:**
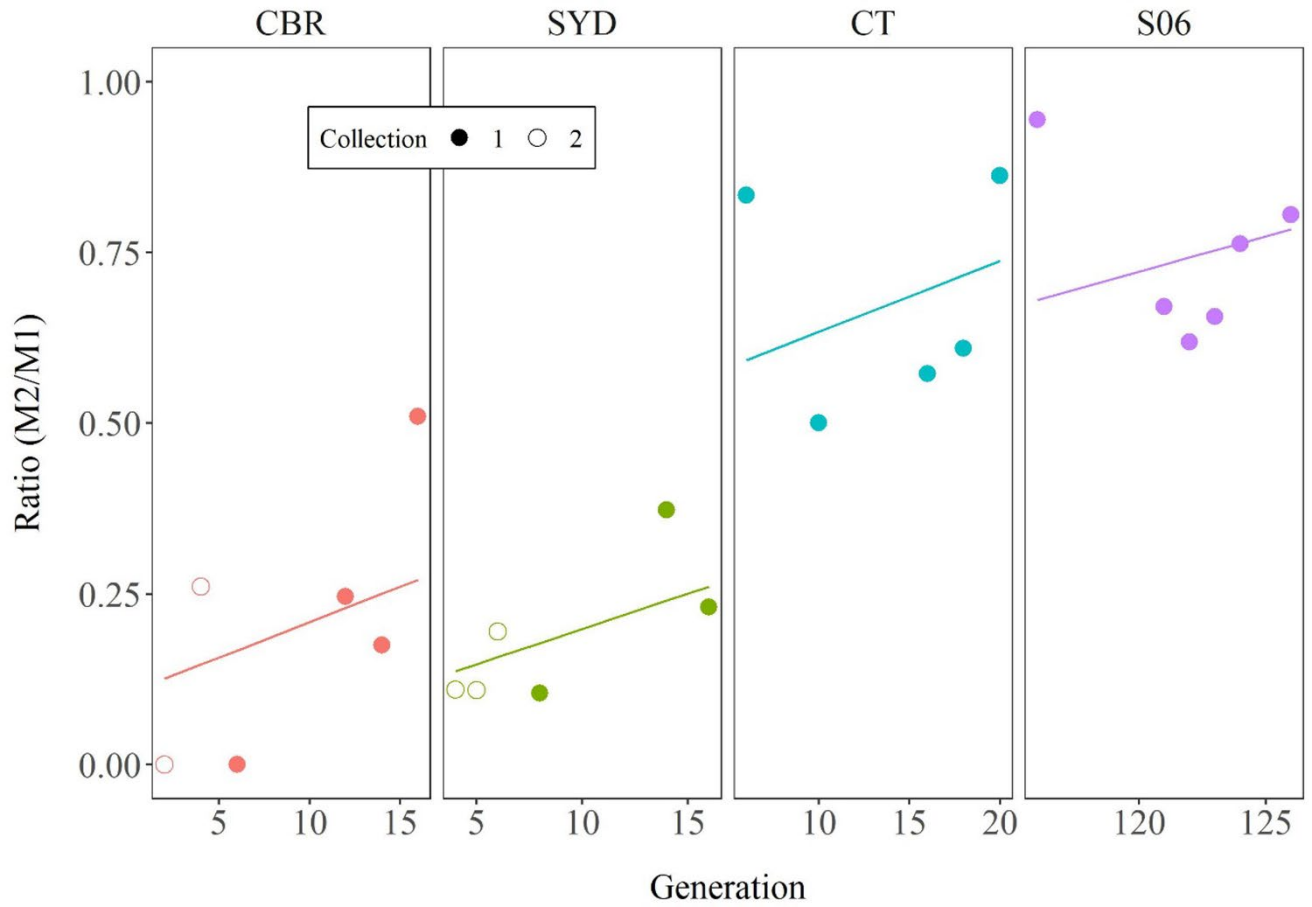
Changes in the M2%/M1% ratio for the three wild/caught populations and S06 over generations in Experiment 1. The data for different populations and the -1 and -2 SYD and CBR collections are shown with different colours and symbols respectively, as per Fig. 3. Lines of best fit for each population across generations are also shown.

Plotting M1% against M2% across all population-generation combinations confirmed a strong positive correlation between these two variables (Fig. 3; r = 0.87 and 0.73 with and without the S06 data respectively, *p* <  < 0.001 in both cases), suggesting that some of the variation in remating frequencies between strains and over time reflected the same underlying behaviours as were driving the initial mating frequency differences. However, this plot also showed some displacement of the correlations between the four populations, and plots of the ratio of M2% to M1% (Fig. 4) confirmed that some of the differences between populations and increases over time in M2% persist even when the variation in M1% is taken into account.

Experiment 1 demonstrates heritable variation in both M1% and M2% between populations and increases in both M1% and M2% through domestication. The latter result also implies that there was heritable variation in the source populations on which selection could act during domestication. While the overall correlation between M1% and M2% suggests that much of the genetic variation in the two measures may be the same, the analysis of their ratios indicates some additional genetic effects on M2%.

Whereas the comparison of absolute values of M1 and M2 mating frequencies in Fig. 2 showed the drop in M2 compared to M1 only varied from about 10% to 40%, depending on strain and generation, the M2%/M1% ratios in Fig. 4 show that in relative terms, the likelihood of remating varied over a much greater range. On average, it was less than 20% and about 25% of the first mating rate in the early and late generations respectively of CBR and SYD, but it was just under 70% for the early CT generations and about 75% for the later CT generations and S06. Conversely, remating inhibition was relatively high, at least 80%, in the early CBR and SYD generations but barely a third of that in later CT generations or S06.

### Experiment 2: intra vs inter-strain pairings, excluding reciprocals

Given the evidence for genetic effects on remating frequency, the aim of the next two experiments was to determine whether these effects were mediated by variation in the female or the first or second male. The specific goal of Experiment 2 was to separate the effects on remating due to the first pairing from those due to the second male (Fig. 1B). This experiment focused on the three strains originally collected from Sydney, SYD-1, SYD-2 and S06, because they covered most of the range of mating frequencies seen in Experiment 1. They were tested at G5 for SYD-2, G14 for SYD-1 and ~ G122 for S06, using a protocol that differed from Experiment 1 in two ways. Firstly, M1% was estimated for three groups of females from each strain, one group with males from each strain. Secondly, the females that had mated with males of their own strain at M1 were again divided into three groups at M2, with one group again being paired with males from each strain.

**Figure 5:**
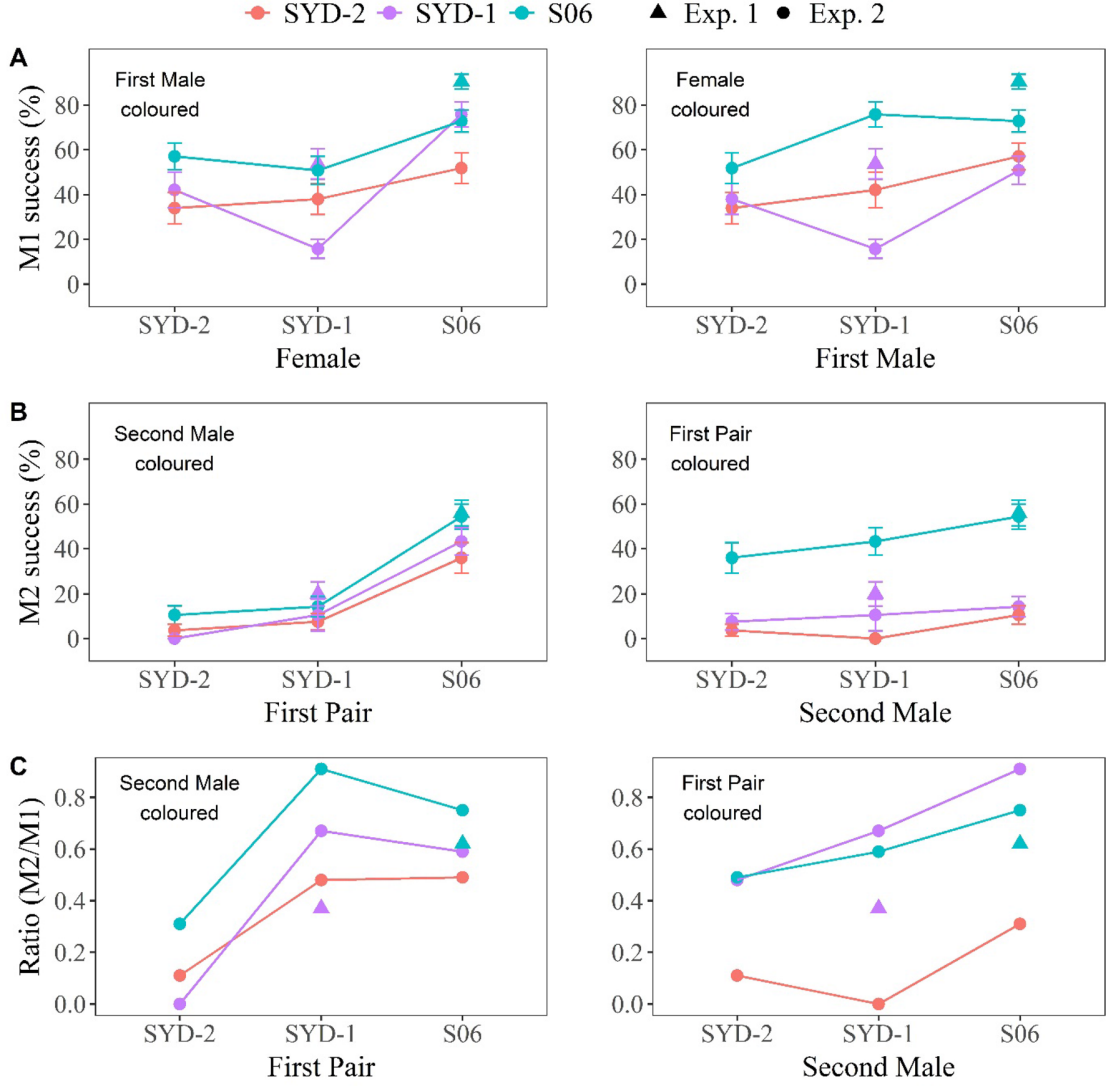
M1%, M2% and M2%/M1% for intra- vs inter-strain pairings, excluding reciprocals. **(A)** M1% ± standard error (se) for intra- and inter-strain pairings of SYD-2, SYD-1 and S06. **(B)** M2% ± se for females previously mated in intra-strain M1 pairings of each of those strains with M2 males of each of those strains. **(C)** M2%/M1% ± se for the females in Panel B. To facilitate visualisation of the effects of each of the different M1 and/or M2 variables, the data for each panel are repeated on two separate graphs, one with each of the other variables on the X axis. Data for the two generations scored have been pooled in all three panels. Where available, results for equivalent pairings and generations from Experiment 1 are also indicated in each panel. Sample sizes for Experiment 2 were 38–68 and 53–81 for intra- and inter-strain pairings respectively for M1 and 35–67 and 19–89 for intra- and inter-strain pairings respectively for M2. Data from Experiments 1 and 2 are shown as triangles and circles respectively.

The results for the intra-strain first matings confirmed the trend of Experiment 1, insomuch as M1% was higher for the domesticated S06 strain than the most recently collected SYD-2 strain (albeit values for the intermediate SYD-1 strain were more scattered) (Fig. 5A). The picture is less clear when inter-strain first matings are considered, likely in part because the sample sizes for those matings were smaller (see Materials and methods). This may explain the against-the-trend drop in the values for S06 males with the more domesticated SYD-1 than the less domesticated SYD-2 females. However, the overall picture is for the first mating propensity of both sexes to increase over the course of domestication. Fitting a quasibinomial GLM (dispersion = 5.47) showed the increase in M1% with generation number was significant for females (estimate: 0.37, t = 2.28, df = 6, *p* < 0.05) and bordered on significance for males (estimate: 0.28, t = 1.78, df = 6, *p* = 0.06).

The M2% data for intra-strain matings (Fig. 5B) also bore out the trend in Experiment 1 for values to increase progressively with domestication. The M2% data for the inter-strain matings also showed broadly similar patterns to those for M1% in Experiment 2, although, as per Experiment 1, values were generally lower than the corresponding M1%s. Again, the smaller sample sizes of the inter-strain pairings make the picture somewhat less clear in those data, exacerbated in this case by the lower remating frequencies overall (e.g., the zero M2% for SYD-2 females with SYD-1 males was only based on 35 pairings). Overall, there is a clear trend for remating frequency to increase with the degree of domestication of both the first pairing (logistic regression estimate: 0.87, z-value = 2.28, *p* <  < 0.001) and the second male (estimate: 0.25, z-value = 2.85, *p* <  < 0.001).

The results for M2%/M1% (Fig. 5C) also showed values increasing with the degree of domestication of the first pair (estimate: 0.12, t = 2.02, df = 6, *p* < 0.05), but in this case not of the second male (estimate: 0.09, t = 1.62, df = 6, *p* = 0.08).

Experiment 2 thus revealed genotypic effects on remating frequencies after controlling for first mating frequencies due to the first pairing but not the second male, whereas genotypic effects on the remating frequencies that were correlated with first mating frequencies were mainly due to both the first pairing and the second male.

### Experiment 3: intra- vs inter-strain pairings, including reciprocals

The primary aim of this experiment was to determine whether the effects of the first pairing on female remating frequencies seen in Experiment 2 were due to male or female influences. A secondary aim was to further test for effects of the second male. This experiment only used the SYD-2 and S06 strains but all four possible pairings involving them were set up for M1 and mated females from each of these four pairings were again paired with males of each strain, resulting in eight combinations (Fig. 1C). Otherwise, the protocol was the same as for the other experiments.

**Figure 6:**
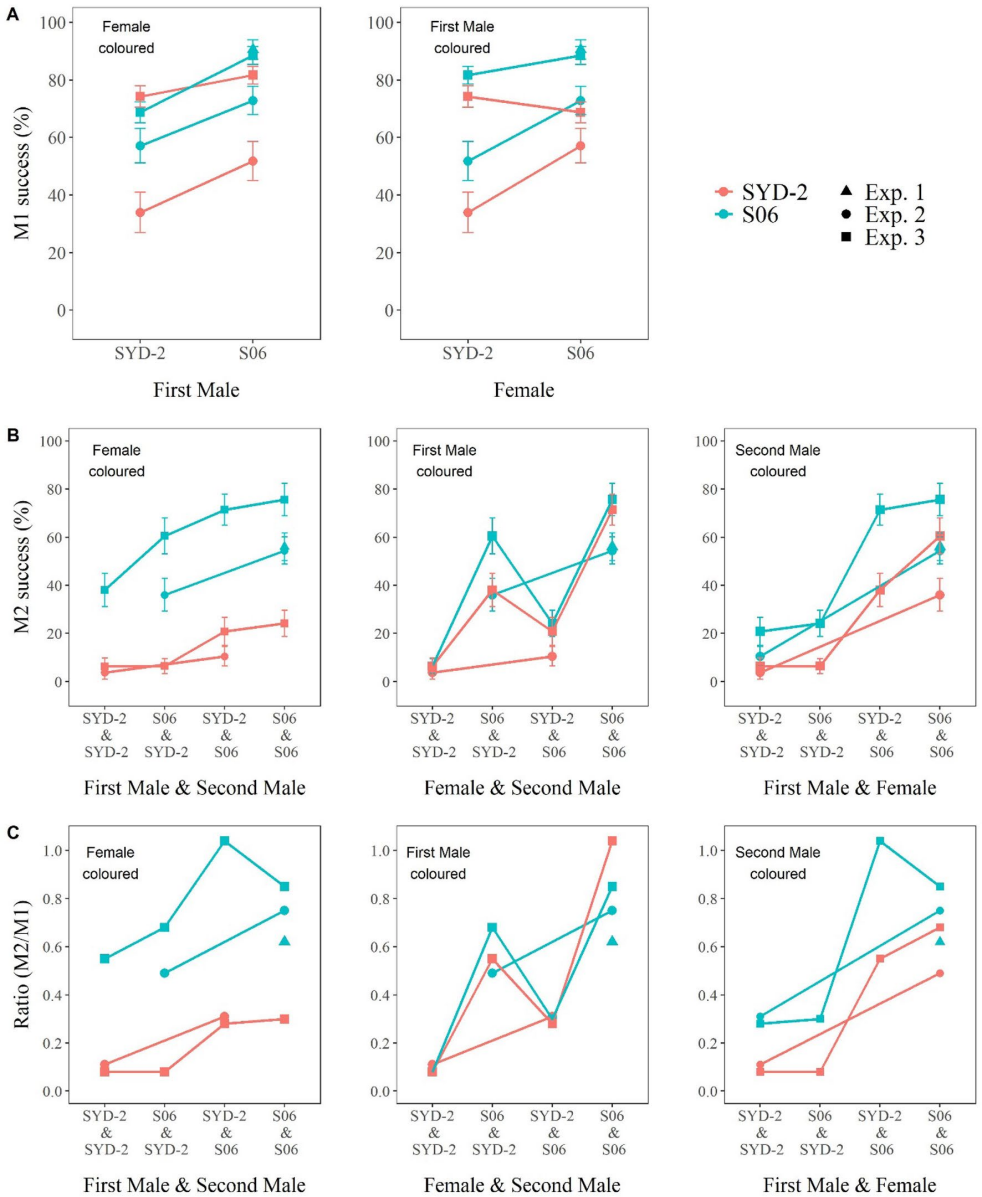
M1%, M2% and M2%/M1% for intra- vs inter-strain pairings, including reciprocals. (**A)** M1% ± se for intra- and reciprocal inter-strain pairings of SYD-2 and S06. **(B)** M2% ± se for females previously mated in all the M1 pairings with M2 males of each strain. **(C)** M2%/M1% ± se for the females in Panel B. To facilitate visualisation of the effects of each of the different M1 and/or M2 variables, the data for each panel are repeated on two (**A**) or three (**B**, **C**) separate graphs, one with each of the other variables (**A**) or combinations of variables (**B**, **C**) on the X axis. Data for the two generations scored have been pooled in all three panels. Where available, results for equivalent pairings and equivalent or adjacent generations from Experiment 1 are also indicated in each panel. Sample sizes for Experiment 3 were 136–160 for M1 and 41–75 for M2. Data from Experiments 1, 2 and 3 are shown as triangles, circles and squares respectively.

The M1% results from this experiment concurred with those of Experiment 2 in indicating higher first mating propensity in S06 as compared to SYD-2 males with either S06 or SYD-2 females (Fig. 6A). Anomalously, however, it did not detect the same difference in first mating propensity of S06 vs SYD-2 females as in Experiment 1, particularly in pairings with SYD-2 males. This difference was reflected in a strong interaction effect of female and first male generation number in the quasibinomial logistic regression model (estimate = 0.10, z = 2.55, *p* < 0.05). One-sided 95% confidence intervals (CIs) for first male generation effects were always positive regardless of female generation (0.04 to ∞ when mated to SYD-2 females, and 0.29 to ∞ when mated to S06 females). In contrast, one-sided 95% confidence intervals for female generation effects were positive when mated to S06 males (95% CI 0.04 to ∞) but not SYD-2 males (95% CI −0.20 to ∞). We suspect this anomaly may have been due to batch differences in females; we note that Experiments 2 and 3 were of necessity conducted on different, albeit near-adjacent generations, and there was significant variation in M1% among the early generations of SYD-2 (Fig. 2). The fact that first mating frequencies were higher overall in Experiment 3 than Experiment 2 also suggests batch effects.

Since M1% showed little difference between females of the two strains, M2% and M2%/M1% showed similar patterns of differences between groups (Fig. 6B, C). As in Experiment 2, both measures showed a large and consistent effect of female genotype, with higher values for S06 than SYD-2 females in all comparisons (M2% trend estimate: 0.85, z = 9.16, p <  < 0.001; M2%/M1% trend estimate: 0.21, t = 9.17, df = 4, *p* <  < 0.001). In this case there was also a smaller but still significant effect of second male genotype on both measures, again with higher values for S06 than SYD-2 (M2% trend estimate: 0.43, z-value = 4.74, *p* <  < 0.001; M2%/M1% trend estimate: 0.09, t = 4.16, df = 4, *p* = 0.007). However, the only effect of the first male was a greater value for S06 than SYD-2 first males mated with S06 females that were subsequently mated with SYD-2 males, which was significant for M2% (Fig. 6B; trend estimate: 0.15, z = 1.75, *p* < 0.05) but not M2%/M1% (Fig. 6C; estimate: −0.003, t = −0.15, df = 4, *p* = 0.4).

Experiment 3 demonstrates that the effects of the first pairing genotype on remating frequencies in Experiment 2 were largely due to differences in females. Experiment 3 also reinforced the evidence from Experiment 2 for an effect of the second male genotype. However, there was only limited evidence from this experiment for an effect of the first male genotype on remating.

## Discussion

We have found significant differences in both M1% and M2%, as well as the M2%/M1% ratio, between recently collected Qfly strains from three regions. In particular, M1% was relatively low in strains from Canberra (CBR) in the early generations of domestication (G2–G6), while both M2% and M2%/M1% were relatively high in the strain from Cape Tribulation (CT). Geographic variation in morphological and climate stress response traits and genetic and genomic markers have been reported in Qfly^63,67,68^, but we are unaware of any previous work investigating geographic variation in remating traits. Collections from Canberra, Sydney and Cape Tribulation have each been differentiated in other traits/markers, so our findings reinforce the overall patterns of ecotypic variation between these populations. The variation we found was relatively large; for example, the Cape Tribulation strain showed about three-fold less remating inhibition than the other two populations. The fact that the differences involve the Canberra and Sydney populations is notable because these populations appear only to have been established over the last eighty years, as part of the southward expansion of the species associated with intensive irrigated horticulture^69,70^.

Relatively few studies have screened for geographic differences in reproductive behaviours in other tephritids, and none to our knowledge have tested for differences in remating inhibition. However geographic variation has been described for first mating frequencies and courtship behaviours in *C. capitata* and *B. dorsalis*, as well as for male sex pheromone composition in *C. capitata* and *A. fraterculus*^71–75^*.* Given the many well-documented cases of differences in reproductive phenotypes among sibling tephritid species, it might be expected that population differences in reproductive phenotypes would also occur within tephritid species^64,76,77^.

There is a larger literature on genetic changes in mating and remating frequencies associated with domestication in tephritids, including some on Qfly. Of particular relevance here, studies of *B. dorsalis*, *A. ludens* and *C. capitata* have found, as in the present study, that the level of remating inhibition declines during domestication^51,56,57^. On the other hand, the one precedent for Qfly, by Pérez et al.^62^, did not find this pattern. That study compared two strains from the same locality (Brisbane), one after five and the other after 49 generations in the laboratory. They found the latter strain showed greater first mating propensity in both sexes, which is consistent with our findings, plus longer female copulation duration and greater sperm transfer by males, but no clear difference in remating inhibition. The discrepancy might in part be due to differences in testing regimes, but it might also be relevant that the domesticated strain used in the present study (G116-126) had been in the laboratory over twice as long as that of Pérez et al. (G49) and the changes we saw over the 12–14 generations in which we screened our three recently collected strains were relatively small and subject to significant fluctuation in individual generations. On average the differences we saw in M2%/M1% over the course of the 12–14 generations within each population were only ca. 10%, whereas the differences we saw between the three recently collected populations and between two of them and S06 were ca. 50%.

The fact that we observed changes in remating inhibition during the domestication in strains from all three source populations implies that there was genetic variation for the trait within each of these populations in addition to the differences between them. This suggests that the demographic factors affecting the optimum reproductive strategies of either or both sexes are also variable both within and between populations. This might be expected given the high level of seasonality and spatial heterogeneity in population numbers^63,67,78,79^. Interestingly, our tropical Cape Tribulation population would be exposed to very different seasonal climate variation from those experienced by our temperate Sydney and Canberra populations, and the Cape Tribulation population differed from the other populations in terms of its (lower) level of remating inhibition.

Given this latter finding, it would now be interesting to screen other latitudinally widespread tephritid species to see whether they show similar differences between populations. In this respect we note that inter-specific comparisons in a wide variety of animals by Taylor et al.^80^ showed higher levels of polyandry at higher latitudes, which could reflect lower levels of remating inhibition. The direction of this latter difference is thus opposite to that we see in our intraspecific analysis. However, it is unclear to what extent the same factors would operate within Qfly as underpin the wide interspecific differences reported by Taylor et al.^80^.

All the strains used in our inter-strain mating trials were from Sydney. They thus provided insight into the basis of the differences due to domestication but not the geographic variation evident in our intra-strain comparisons. Specifically, they showed that the genotype of the female and, to a lesser extent, the second male contributed to the changes that occurred during domestication, with only equivocal evidence for a first male effect. We were surprised not to find a clearer effect of the first male genotype on female remating inhibition in these trials because experiments injecting seminal fluid into virgin females, and experiments with aspermic males, have clearly shown that Qfly semen does inhibit mating^50,59^. Moreover it does it in a complex, dose-dependent way which does not seem to depend on the amount or quality of sperm^54,59,62,81^. While *Bactrocera* genomes apparently do not contain genes encoding orthologues of either the sex peptide, which mediates sperm-independent remating inhibition in *D. melanogaster*, or its close homologue DUP99B^37,82^, it is expected that some component(s) of the seminal fluid other than sperm contributes to this dose-dependent effect^32,83–85^. It may be that there was simply too little genetic variance for the relevant component(s) within the Sydney source population to generate a clear selective response to domestication.

We suspect that at least some of the effect of female genotype on remating frequency in our intra-strain mating trials reflected variation in general receptivity to mating that was operative in both first and second matings. This would explain the significant correlation between first and second mating frequencies seen through domestication (and in fact also across strains from different localities). Clearly many aspects of female mating behaviour would be common to the two events; aspects that are readily measurable and found to vary in various higher Diptera include mating latency, copula duration, fecundity and sperm competition, with mating latency also one which has been found to vary between strains of other tephritids^19,72,86,87^. However, it is also possible that second mating-specific differences are involved. We note that the correlation with female first mating frequencies across the whole data set only explained about 76% (or 53% excluding the S06 data) of the variance in second mating frequencies. While there may not have been genetic differences between the recently collected and domesticated Sydney strains in the quantity of the males’ seminal fluid component(s) that trigger remating inhibition, there could well have been genetic differences between those strains in the females’ receptivity to the trigger(s).

The effect of the second male genotype on remating frequency which we saw in our inter-strain trials was in effect a first mating effect for those males, since most of them were virgins and those that were not had been separated from females before their use long enough to fully replenish their sperm stores and recover their mating propensities (see Materials and methods). As such, male as well as female genotype may have contributed to the differences in first mating frequencies we observed between domesticated and recently established strains in our intra-strain trials. This is also consistent with the greater engagement in courting and mounting behaviours of domesticated Qfly males as compared to those from recently established colonies, and their higher levels of sex pheromone emissions^88–90^.

The effectiveness of SIT programs depends heavily on, firstly, the ability of the sterile males to compete successfully for matings with field females and, secondly, on their ability to then inhibit those females from remating with fertile field males. At face value, our finding that remating inhibition declines during domestication suggests that SIT programs should aim to use strains that have been domesticated as little as their mass rearing procedures allow. However, our finding that little of the effect was due to M1 male genotype argues against this. Indeed, the fact that M2 male genotype was important to female remating frequency and changes in M2% during domestication were positively correlated with changes in M1% might suggest that males of domesticated strains might at least compete better than those of recently established strains for matings with field females. Also complicating the issue is the fact that the technology to produce male-only mass reared flies for SIT purposes has not yet been developed for Qfly, so the increased remating propensity of domesticated females means the effect of the sterile males released on the wild population is further diluted by them also mating with the co-released sterile females. Whatever the net effect of domestication on the mating and remating behaviour of the mass released strains, the success of the SIT program may be more dependent on other characters, such as the reduced climate stress resistance and, by inference, robustness of domesticated strains under field conditions^78^. Perhaps our findings of most relevance to SIT are that considerable genetic variance for mating and remating propensities exist both within and between source populations, so careful selection of source population and rearing conditions that select for high mating propensities for males but not females could materially enhance the efficacy of the release programs.

## Materials and methods

### Insect strains and culture conditions

Five strains collected relatively recently from three different source populations were used in this study, one from Cape Tribulation (CT) and two each, collected several months apart, from Sydney (SYD-1 and SYD-2) and Canberra (CBR-1 and CBR-2). The long domesticated S06 strain^63,78^, also originally collected from Sydney, was used as the control strain for all bioassays. Details of the six strains are in Table 1.

**Table 1:** Qfly source populations and strains used in this study.

Source population	Location	Approximate coordinates	Source fruit	Time of year (season)	Year (strain)	Initial larval diet	Bioassay generations
CT	Cape Tribulation	16.09°S; 145.46°E	Carambola	Aug (Dry)	2018 (CT)	Baby (vine) capsicum and gel diet (G0-G4)	G6, G10, G16, G18, G20
CBR	Canberra	35.27°S; 149.11°E	Apricots and peaches	Jan (Summer)	2019 (CBR-1)	Baby (vine) capsicum and gel diet (G0-G4)	G6, G12, G14, G16,
				Feb (Summer)	2020 (CBR-2)	Gel diet	G2, G4
SYD	Sydney	33.9°S; 151.14°E	Loquats	Sep (Spring)	2019 (SYD-1)	Baby (vine) capsicum and gel diet (G0-G4)	G8, G14, G16
				Jan (Summer)	2020 (SYD-2)	Gel diet	G2, G4, G6
			N.A.	Late spring	2006 (S06)	Carrot diet	~ G116-G126

Culture conditions were as described in Popa-Báez et al.^63^ except for some changes to the diet. The first lines collected in SYD and CBR were initially reared on baby capsicum diet for four generations before being transferred to gel diet^91^, whereas the second two lines from these locations were cultured on gel diet from the outset and S06 had been raised for many generations on the carrot diet before being transferred to gel diet about ten generations before our experiments began. All lines were reared on gel diet for at least two generations before our experiments began.

In accord with our previous findings with recently collected strains, we found flies from our four newly established strains were sexually mature at 12–14 days post-emergence^62,92^. Accordingly, we used flies that were 16–18 days post-emergence when first tested in mating experiments. The S06 flies reached sexual maturity by 6–8 days and were tested 8–12 days post-emergence. Virgins for the experiments were separated into sexes within 3–4 days of emergence and the separate-sex cages in which they were then kept were checked before use to ensure they contained no opposite sex flies.

### Experiment 1: intra-strain first and second pairings

In this experiment virgin females from each strain were first paired individually with a virgin male from the same strain and those females that mated were then given a second opportunity to mate with a (different) virgin male from the same strain four days later (Fig. 1A). Preliminary experiments with two, four- and eight-day intervals between first and second matings showed no consistent difference in remating frequencies between pairings after these intervals (Fig. S1), although results were somewhat more variable after eight-day intervals. Batches of up to 350 pairings were tested at a time, using several observers to ensure accurate monitoring of mating.

The scale of the experiment meant that we could generally only test four of the six strains in each batch. We therefore generally only included one each of the two SYD and two CBR strains in each batch. However, the staggered collection times for these strains enabled us to test all three source populations at five or six generations across their first 15–20 generations in the laboratory. The S06 control strain (generations 116–126 over the course of the experiment) was included in all batches of tests, which enabled us to check for any batch effects.

Experiments were conducted in a dedicated room maintained at 25 ± 1 °C, 65 ± 5% relative humidity, with a 1:11:1:11 h dawn: day: dusk: night cycle. Lighting was ca. 2000 Lux during the day and < 100 Lux at dusk and dawn on the benches where the pairings were set up. First pairings were set up about two and a half hours prior to dusk by placing a single pair in a 425 ml transparent plastic cup (Cat. No. 72205; Dowlings Canberra) containing a small sponge and covered with a transparent dome-shaped lid. No food was provided because Qfly mating is a non-resource-based system^93^.The timing was chosen because Qfly mating activity is triggered by the onset of dusk and matings continue for a variable period thereafter, including in darkness^66,92^.

The cups containing potential mating pairs were monitored continuously from the onset of dusk until 1.5 h of darkness had elapsed (using a LED torch covered with a red filter during darkness). Mating was considered to start with intromission and end when the pair separated. Females that sustained an uninterrupted copulation for 30 min or more were considered mated. Preliminary experiments (Fig. S2) showed that copulations lasting less than 30 min had lower level of mating inhibition than those of 30 min or longer. Once matings had been completed, the females of each line were transferred to a 10 L rearing cage (BugDorm-4 M insect cages, Cat. No. 4M1515, BugDorm Store) with provision of water, sugar and yeast hydrolysate and kept under standard laboratory conditions until their second pairing.

Second mating frequency was measured using the same protocol as the first except that the flies used were four days older. On a few occasions when it was necessary to use non-virgin males in the second pairing, males from the first pairing were used; an interval of four days without mating is sufficient for sperm stores and mating frequencies of once-mated males to be fully restored to virgin levels^47^.

### Experiment 2: intra- vs inter-strain pairings, excluding reciprocals

This experiment used only the three Sydney strains, i.e., SYD-1, SYD-2 and S06, and these were tested in two batches, one involving SYD-1 at G14 and S06 at G122, and the other involving SYD-2 at G5 and S06 at G124, respectively. Thus, they ranged from recently collected to intermediate and long-domesticated populations, respectively. Test conditions were as per Experiment 1, with two exceptions. Firstly, three groups of females from each strain were tested in the first pairing (M1), one group being paired with males from each of the three strains. Secondly, the females that had mated with males of their own strain at M1 were again divided into three groups for M2, one group paired with males from each of the three strains (Fig. 1B).

### Experiment 3: intra- vs inter-strain pairings, including reciprocals

This experiment only used SYD-2 and S06 and tests were carried out in two batches, on generations SYD-2 G5 and S06 G122 and SYD-2 G7 and S06 G124 respectively. In this case both reciprocal pairings between the two strains, plus the two intra-strain pairings were set up for M1. Then for M2, mated females from each of these four M1 combinations were again paired with males from one or other of the two strains, resulting in eight combinations in total (Fig. 1C). Otherwise, the experimental protocol was the same as for the first two experiments.

### Statistical analysis

All statistical analyses were performed in R (v4.0.2), within RStudio IDE (v. 1.4.1103). We initially fitted linear models to all three response variables (M1%, M2% and M2%/M1%). Where assumptions were violated (for both M1% and M2%), we proceeded to fit a generalized linear model (GLM) for distributions in the binomial family. If overdispersion was detected, GLM was fitted for distributions in the quasibinomial family.

For Experiment 1, the models for M1%, M2% and M2%/M1% incorporated population as a fixed effect and the natural logarithm of generation number as a continuous variable, plus their interaction. Using the car package^94^, Type 3 analysis of deviance (ANODEV) and analysis of variance (ANOVA) were performed on the models to test the significance of the effects of population, generation number and their interaction. Where interaction terms were not statistically different from zero, the model was refitted without the interaction terms. Pearson correlation coefficients between M1% and M2% were also calculated.

Trends over generation number established in Experiment 1 were used to modify alternate hypotheses in Experiments 2 and 3, i.e., changing from two-sided to one-sided tests. For both these latter experiments models of M1% were fitted against the natural logarithms of first male generation number and female generation number. M2% and M2%/M1% were fitted against natural logarithms of first pair and second male generation numbers in Experiment 2, and against natural logarithms of first male, second male and female generation number in Experiment 3. When interaction terms were not significant (and thus removed), generation trends were assessed with t-tests (if using linear model or quasibinomial GLM) or z-tests (binomial GLM, logistic regression). When the interaction was significant, estimated marginal means of linear trends (emtrend) were assessed and one-sided 95% confidence interval were computed using the emtrend function from the emmeans package^95^.

Displays were generated using ggplot2 in the tidyverse package^96^ and the ggrepel^97^ and ggpubr^98^ packages.

## Supplementary Information

Supplementary Information.
