# Supplementary material for: Population differences and domestication effects on mating and remating frequencies in Queensland fruit fly
Source: Sci Rep. 2022 Jan 7;12:153. doi: 10.1038/s41598-021-04198-4 (PMC8741809; doi:10.1038/s41598-021-04198-4)

**Table S1.** M1% mating frequencies and their standard errors for the two cohorts of females for various strain-generation combinations. The data for the second cohort was always used in the analyses because it was collected on the same day and on similarly aged females as the corresponding M2% data. M1% data for the first cohort was not always collected and never used because it was collected four days earlier on four-day-younger females. On a few occasions there were significant differences between the two estimates, although there was no consistent direction to the differences. Two cases in which the same generations were tested in Experiments 1 and 2 are indicated with asterisks (* for SYD-1 G14 and ** for S06 G122).

| **Exp** | **Strain** | **Generation** | **First cohort M1 (% ± SE)** | **Second cohort M1 (% ± SE)** |
| --- | --- | --- | --- | --- |
| 1 | CBR-2 | 2 | 37.0 ± 2.8 | 16.0 ± 4.4 |
| 1 | CBR-2 | 4 | 27.1 ± 3.1 | 28.1 ± 5.6 |
| 1 | CBR-1 | 6 | 13.3 ± 2.2 | 10.4 ± 3.1 |
| 1 | CBR-1 | 12 | 28.5 ± 3.2 | 31.9 ± 5.5 |
| 1 | CBR-1 | 14 | 54.7 ± 4.3 | 46.9 ± 3.4 |
| 1 | CBR-1 | 16 | 50.0 ± 6.8 | 58.3 ± 8.2 |
| 1 | SYD-2 | 4 | 55.8 ± 3.1 | 49.5 ± 5.2 |
| 1 | SYD-2 | 6 | 64.4 ± 4.7 | 58.7 ± 6.2 |
| 1 | SYD-1 | 8 | 35.8 ± 3.1 | 43.8 ± 8.8 |
| 1 | SYD-1^⁎^ | 14 | 15.0 ± 2.8 | 15.8 ± 4.2 |
| 1 | SYD-1 | 16 | 56.5 ± 4.8 | 62.9 ± 3.7 |
| 1 | CT | 6 | 45.8 ± 2.5 | 37.5 ± 8.6 |
| 1 | CT | 10 | 46.9 ± 3.6 | 50.0 ± 8.8 |
| 1 | CT | 16 | 72.0 ± 4.5 | 80.9 ± 4.8 |
| 1 | CT | 18 | 68.8 ± 5.3 | 65.7 ± 4.1 |
| 1 | CT | 20 | 54.8 ± 6.3 | 62.1 ± 6.4 |
| 1 | S06 | 111 | 60.8 ± 3.3 | 87.5 ± 5.9 |
| 1 | S06 | 116 | 78.9 ± 2.9 | 81.3 ± 6.9 |
| 1 | S06 | 121 | 83.3 ± 3.0 | 84.9 ± 4.2 |
| 1 | S06^⁎⁎^ | 122 | 78.0 ± 4.1 | 90.5 ± 3.4 |
| 1 | S06 | 123 | 89.3 ± 3.0 | 78.7 ± 5.2 |
| 1 | S06 | 124 | 85.9 ± 3.9 | 85.3 ± 3.1 |
| 1 | S06 | 126 | 83.3 ± 4.6 | 93.2 ± 3.8 |
| 2 | SYD-2 | 5 | 44.1 ± 2.7 | 34.0 ± 6.5 |
| 2 | SYD-1^⁎^ | 14 | 51.7 ± 2.6 | 53.7 ± 6.8 |
| 2 | S06^⁎⁎^ | 122 | 72.9 ± 2.4 | 72.8 ± 4.9 |

**Figure Legends**

**Figure S1.** M1% and M2% values and their standard errors for three strains with two-, four- and eight-day intervals between the two pairings. Sample sizes were 32 for M1 and 16-36 for M2.

**Figure S2.** M1% values for intra-strain S06 pairings and M2% for intra-strain S06 pairings in which M1 had been interrupted 10 or 20 minutes or uninterrupted (> 30 minutes) after stable joining. Sample sizes were 72-152.

Figure S1


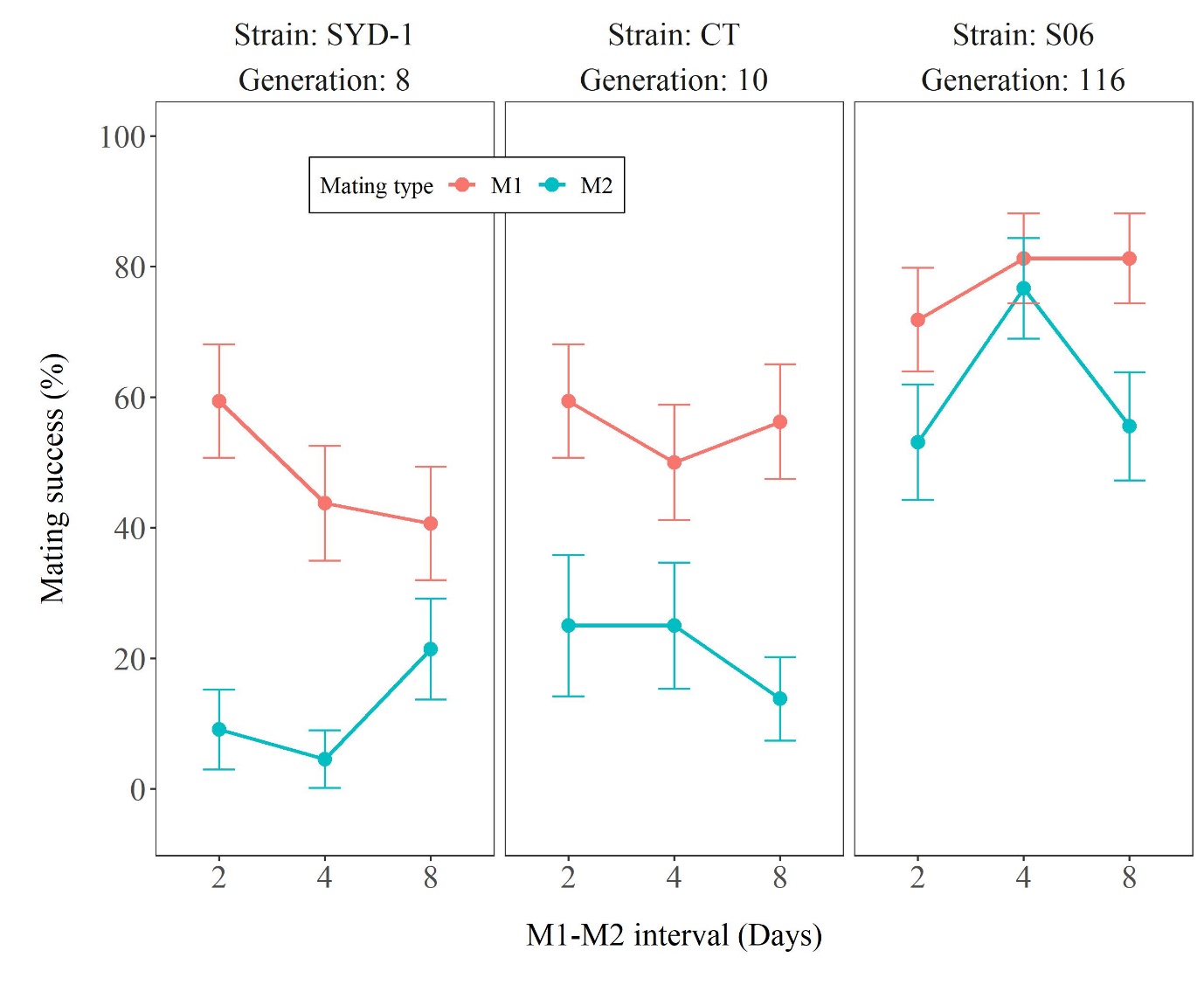


Figure S2
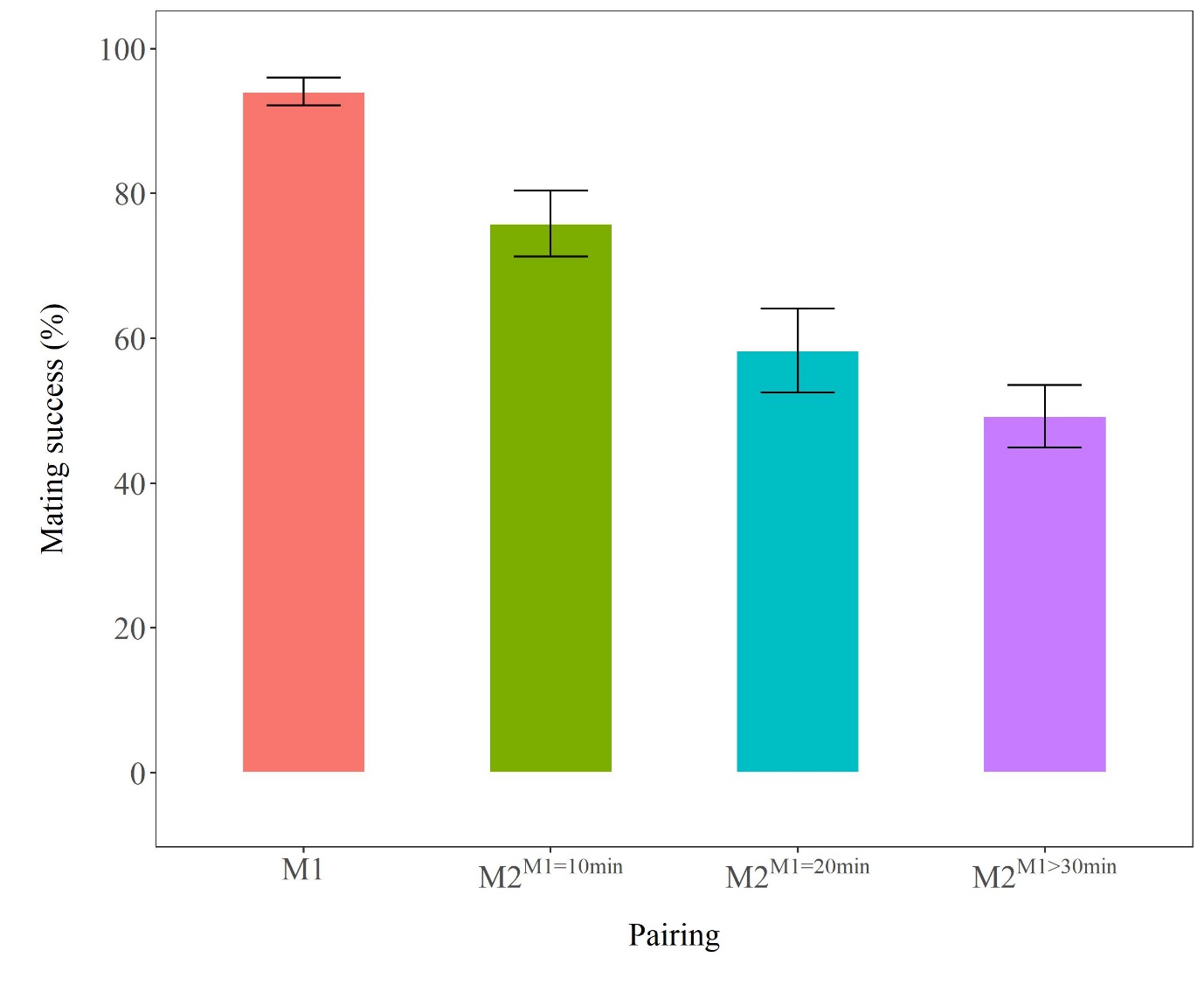

Supplement: Supplementary file 1 — Supplementary Information. [file 41598_2021_4198_MOESM1_ESM.docx]
